# Focus on the Narrative Skills of Teenagers With Developmental Language Disorder and High Functioning Autism

**DOI:** 10.3389/fpsyg.2021.721283

**Published:** 2021-10-26

**Authors:** Lucie Broc, Elise Brassart, Anne Bragard, Thierry Olive, Marie-Anne Schelstraete

**Affiliations:** ^1^Université de Poitiers, Université de Tours and CNRS, Tours, France; ^2^Université Côte d’Azur and CNRS, Nice, France; ^3^Université Catholique de Louvain, Louvain la Neuve, Belgium

**Keywords:** developmental language disorder (DLD), high functioning autism (HFA), narrative skills, teenagers (adolescence), spoken language

## Abstract

**Purpose:** Narratives of personal experiences emerge early in language acquisition and are particularly commonly experienced in children’s daily lives. To produce these stories, children need to develop narrative, linguistic, and social-cognitive skills. Research has shown that these skills are impaired in children with developmental language disorder (DLD) and high functioning autism (HFA).

**Aim:** This study aimed to determine whether narrative skills are still impaired in adolescence and to highlight the language similarities and differences between teenagers with DLD and HFA in the production of a narrative of a personal experience.

**Method:** Ten teenagers with DLD, 10 teenagers with HFA and 10 typically developing (TD) teenagers, matched on chronological age, told a narrative of a personal experience. These stories were analyzed to evaluate narrative skills through coherence (respect of the narrative schema) and cohesion (anaphora and connectors) and social-cognitive skills (affective and cognitive mental states of the characters, and arbitrary vocalizations such as voice noises).

**Results:** Teenagers with DLD were less compliant with the complication step in the narrative schema than teenagers with HFA or TD. No difference was observed between the three groups of teenagers in terms of cohesion or regarding the positive and negative social-cognitive skills used in narratives.

**Conclusion:** When producing a narrative of a personal experience, HFA teens do not have difficulties neither with narrative skills and with social-cognitive skills assessed in this paper. In DLD the profile of the teens is not the same: They do not have difficulties with social-cognitive skills and with a part of narrative skills (cohesion), and they have difficulties with the narrative schema.

## Introduction

Developmental language disorder (DLD) and high functioning autism (HFA) are classified as neurodevelopmental disorders in the 10^*th*^ International Classification of Diseases (CIM10). Children with DLD and HFA experience difficulties with oral language, particularly when they tell a narrative of a personal story (Norbury and Bishop, 2003). Therefore, oral language skills acquisition is often evaluated using a narrative of a personal experience (McCabe and Rosenthal-Rollins, 1994). Describing a personal experience is an ecological skill because it emerges early in language acquisition and is particularly commonly experienced in children’s daily lives (Kern, 1997; Reilly et al., 2004).

Studies conducted in childhood have shown that in children with DLD have significant language difficulties that result in poor narrative performance (Botting and Conti-Ramsden, 2004; Leonard, 2014) and that children with HFA also encounter difficulties in the social use of language (Adams, 2005), which affect their narrative skills (Goldman, 2008). Few studies have investigated the period of adolescence in DLD and in HFA even though studying oral language development during this period in ecological narrative situations could allow for better targeted remediation and intervention. It is therefore important to study the similarities and specificities in oral language skills of teenagers with DLD or HFA when they tell a narrative of a personal experience. Using narratives of personal experiences indeed allows one to measure the narrative skills at the linguistic and social-cognitive levels, as involved in typical language development (de Weck and Rosat, 2003; Norbury and Bishop, 2003) or in neurodevelopmental disorders such as DLD (McCabe et al., 2008) and HFA (Botting, 2002).

### Narrative Skills

A narrative is made up of statements that are interconnected with each other and organized into a coherent whole (Kern, 1997; de Weck, 2004). Constructing a narrative requires the appropriate use of language as a communication tool, which is a question of using language in a given situation and considering the interlocutor and the context of the interaction (Coquet, 2005). Moreover, constructing a narrative requires managing both its coherence, i.e., proposing a story structured in several steps at the temporal, causal and thematic levels (at the level of the overall macrostructure), and its cohesion, which is defined by the creation of links between two statements (at the microstructure level) with the help, for example, of anaphora or connectors (Favart and Passerault, 1999; de Weck and Rosat, 2003). Narrative skills such as coherence and cohesion are acquired between 3 and 8–9 years of age. Twelve-year-old children are comparable to adults in this respect (Fayol, 1985; Godard, 1994; Hilaire-Debove and Kern, 2013). Difficulties with narrative development have been observed in children with DLD and HFA, but they do not manifest in the same way depending on the neurodevelopmental disorder studied (Bruck et al., 2007).

### Coherence

As indicated above, textual coherence refers to the idea that the different statements of a text must present a continuity of meaning to make it semantically meaningful (Halliday, 1975). Coherence can be achieved through syntactic devices and by logical text structures and schema for example. With narratives, coherence depends on the respect of the narrative schema, which is composed of four invariable steps (Fayol, 1985):

1. The initial situation, i.e., the setting of a spatial-temporal event that is described and the presentation of the character(s);
2. The triggering event that disrupts the initial balance;
3. One or more attempts that aim to reestablish the initial balance; and
4. The resolution of the conflict.

Acquisition of the narrative schema is influenced by nature of the narrative that the child produces (Gonnand and Jisa, 2000; Bernicot, 2010). With regard to accounts of personal experiences, it is only from the age of 6 that children are able to independently evoke the successive steps of a narrative, without the need for adult intervention (de Weck and Rosat, 2003).

Some studies conducted with bilingual children on cohesion have shown that there is similar performance between children with and without DLD (Altman et al., 2016; Tsimpli et al., 2016) whereas others reports that children with DLD experience difficulties. de Weck and Marro (2010) showed that from the age of 4, when compared to age-matched peers, children with DLD produce narratives of personal experiences with less information. In addition, adherence to the narrative schema is less comprehensive. Between the ages of 9 and 11, in a storytelling situation focused on a given theme and regardless of the type of story asked of them, children with DLD tend to omit the two central steps of a story, namely, the disruptive element (phase 2) and the attempt to resolve the conflict (phase 3) (Merritt and Liles, 1989). It is important to note, however, that in general, in all children’s narratives, the steps that are least often provided are those related to the conflict resolution phase (phase 3) (de Weck and Rosat, 2003). For example, 11-year-old HFA participants providing personal experience narratives also had difficulty producing coherent narratives, as they have comprehensive knowledge of the narrative schema but consistently produced fewer elements in the core steps than typical participants of the same age (Goldman, 2008). Losh and Capps (2003) also found that in producing a narrative about a personal experience, participants with HFA aged 8–14 were less consistent with the narrative schema than typical participants of the same ages.

Difficulties with producing a narrative that adheres to the narrative schema persist over time in participants with DLD and HFA. Reed et al. (2007) found, for example, the rate of information provided in the narratives of teenagers with DLD of 13 years and 2 months to 15 years and 9 months to remain lower than that of typical adolescents of the same ages. Barnes and Baron-Cohen (2012) assessed the type of information (setting, characters, conflicts, and resolution) displayed in narratives by adult participants with HFA and TD controls. The authors found that the participants with HFA tended to provide local rather than global details, which was not the case for the typical control participants. In the initial situation, for example, adults with HFA provided items such as “*there was another white bed and the lady was blond*” but did not specify that they were in a hospital. On the other hand, Colle et al. (2008) provided a narrative based on a picture book such as “frog where are you?” (Mayer, 1965), and did not observe difference between adults with HFA and their age-matched controls in terms of coherence of the story.

### Cohesion

Cohesion refers to the links between the different statements produced in a narrative (Hickmann, 2004) and to the meaning of what is said (Halliday and Hasan, 1976). To create such links, the speaker uses various linguistic tools, including anaphors and connectors (de Weck, 2004). Connectives and anaphors carry specific functions in discourse processing. As a syntactic function, they link together textual segments, and as semantic and procedural functions, they provide instructions to the addressee to accurately integrate textual content (Favart et al., 2016). Their main role is to operate as processing instructions, in that they instruct the addressee how to connect or separate discourse events and so help him/her to draw up an accurate representation of the overall discourse. Specifically, anaphors maintain the presence of protagonists throughout the narrative by avoiding the repetition of the name already mentioned, which ensures thematic continuity. This anaphoric strategy is acquired at approximately 9–10 years of age (Karmiloff-Smith, 1981). According to Liles (1996), anaphors must be used appropriately for the speaker to easily understand what a linguistic tool is referring to (e.g., “**my friends** were going to play sports when **they** saw the eighth-graders coming out of the change room”).

Connectors mark a relationship of meaning between statements produced in a communication situation (Favart and Passerault, 1999), and their function is to organize and establish relationships between elements of discourse (Schneuwly and Bronckart, 1986). The development of connectors occurs over a long period from age 5 to age 12 (Favart and Passerault, 1999). Before the age of 5, children preferentially use the “and” connector, which mark all types of links between the statements produced (McCabe and Peterson, 1991). From the age of 5, children begin to use connectors such as “after” to mark the sequence of events. Finally, from the age of 9, children are able to make semantic links, such as causal relations (“I stole an eraser because I lost mine”; Kern, 1997).

Few studies have investigated cohesion in participants with DLD or HFA. For example, Kaderavek and Sulzby (2000) have shown that from the age of 4, children with DLD tend to systematically reintroduce the referent rather than maintaining it throughout the narrative, which is not the case for typical children of the same age. In addition, these young children use few connectors. For participants with HFA, Novogrodsky and Edelson (2016) compared twenty-four children and teenagers with HFA (from 6:1 to 14:3) with seventeen TD children and teenagers (from 5:11 to 14:4) in a story relling task. The authors highlighted that participants with HFA produced more ambiguous pronouns in the story-generation task than did the TD children. In adulthood, Colle et al. (2008) studied cohesion. The authors found that participants with HFA used fewer anaphors than typical control participants of the same age, in a picture book-based story situation.

### Comparison of Coherence and Cohesion

Petersen’s (2011) systematic review highlights that the majority of studies of participants with DLD report difficulties with coherence and cohesion in narratives. Gillam and Johnston (1992) found, for example, that in a picture-based storytelling task, cohesion was stronger than coherence in 9-year-old to 11-year and 7-month-old children with DLD: They produced more connectors per utterance than correct sequences of ideas. However, the nature of the task seems to have an impact on the performance of participants with DLD, as their performance varies according to the type of story they are asked to tell. For example, Wetherell et al. (2007) compared the narrative skills of 13- to 15-year-old teenagers with DLD and typically developing teenagers of the same age in two production situations: a personal experience and from a picture book story. The authors observed that, compared to typical teenagers, teenagers with DLD produced fewer errors on formal aspects of language (such as number of different words, syntactic units, or complex sentences) for the personal experience story than for the picture book story. The type of task to be performed therefore impacts the narrative performance of participants with DLD.

### Social-Cognitive Skills Used in Narrative

Several social-cognitive skills are necessary to ensure a quality interaction in a narrative. Particularly, to produce a personal experience narrative, social-cognitive skills such as “acquiring discourse management strategies” and “adapting linguistic forms to the criteria of social interaction” (Ninio and Snow, 1996) or “being able to introduce a topic in such a way that your interlocutor can understand you” and “being able to maintain a conversational topic” (Bowen, 2011) are important. For instance, producing irrelevant linguistic forms such as arbitrary vocalizations (voice noises) does not ensure the quality of comprehension of the narrative because it does not respect Grice’s (1979) principle of cooperation.

Social-cognitive skills are acquired in parallel with a child’s language skills (Kern, 1997). For example, with development, children increasingly take the speaker into account by producing linguistic units designed to attract its attention (see also Bereiter and Scardamalia, 1987 and Kellogg, 2008, for text composition). The discourse is adapted according to the knowledge children share with their interlocutor.

With neurodevelopmental disorders, it is important to distinguish between primary and secondary social-cognitive difficulties (Monfort, 2007). The social-cognitive difficulties of participants with HFA is primary and represent the central deficit of their language disorder (Courtois, 2005). According to the DSM-5 classification (American Psychiatric Association, DSM-5 Task Force, 2013), impairments in social-cognitive skills are one of the diagnostic criteria of the autism spectrum. As a result, while the language produced by participants with HFA is of a good level, it is often poorly adapted to social situations because it is used idiosyncratically (Rogé, 2015). Children with HFA indeed have difficulties to respect the rules of communication, particularly for adapting to the speaker. For example, they frequently produce voice noises that are not related to what is being said (Peeters, 2020). In addition, difficulties with social cognition are present in children with HFA. Brown et al. (2012) compared the number of emotional and cognitive items produced by 10-year-old children with HFA and typical children in two personal experience tasks, one positive and one negative. The results show that regardless of the affective valence of the personal experience, the children with HFA produced fewer emotional and social-cognitive items than typical developing children of the same age.

On the other hand, children with DLD take into account the level of knowledge they have in common with their interlocutor to elaborate their narrative, as do typical children of the same ages. Liles (1996) proposed a story retelling task for children with DLD aged 7–10 and for control children of the same ages: children with DLD and typical control children provided more elements of understanding to their interlocutor when they had no shared knowledge with him. Furthermore, Jullien (2008) found that participants with DLD aged 6–11, when speaking to an interlocutor who did not know the story they were to tell, produced more appropriate anaphors to introduce their characters than when the interlocutor already knew the story. These results are in line with those obtained by Van der Lely (1997) for teenagers with DLD aged 10 years and 2 months to 13 years and 11 months. In a context of unshared knowledge with their interlocutor, the teenagers correctly used anaphors with definite and indefinite determiners. It thus appears that participants with DLD, as typical participants, are able to adapt to the type of narrative proposed to them, as well as to the level of knowledge they have in common with their interlocutor. However, the level of this adaptation in participants with DLD remains below that of typical participants of the same age.

### Aims and Hypotheses

Data regarding the narrative and social-cognitive skills of teenagers with DLD or with HFA is scarce. In this frame, this study investigated the language skills of teenagers with DLD and with HFA. Specifically, these teenagers told a narrative of a personal experience, which assessed in terms of coherence, cohesion, and social-cognitive skills. Comparing the narrative and social-cognitive skills of teenagers with DLD and HFA in an ecological situation of communication can facilitate diagnosis and development of better targeted remediations and interventions. Such an intersyndromic comparison is relevant because it allows to determine the specificities of the studied disorders as well as their similarities. Indeed, DLD and HFA are neurodevelopmental disorders that share commonalities, which can make diagnosis difficult (Craig and Trauner, 2018). Despite typical non-verbal IQs, children with DLD experience difficulties with language (Conti-Ramsden and Botting, 1999) while children with HFA have primary difficulties with the social-cognitive aspects of communication (Williams et al., 2008). Using narratives of personal experiences may therefore help to identify the persistent language difficulties that are specific to the DLD or HFA teenagers (Goldman, 2008; Manolitsi and Botting, 2011). Indeed, language is affected on components related to functional aspects for DLD (Conti-Ramsden and Botting, 1999) but to pragmatic aspects for HFA (Williams et al., 2008).

Accordingly, we hypothesized that the narrative produced by teenagers with DLD and with HFA would differ on the narrative and social-cognitive performance: only teenagers with DLD should have difficulties with coherence and cohesion, whereas only teenagers with HFA would have difficulties at the social-cognitive level.

## Materials and Methods

### Participants

Thirty French and French-speaking Belgian teenagers, all enrolled in school, participated in this study:

- 10 DLD teenagers (7 boys) of 10 years and 9 months to 14 years and 1 month in age (*M* = 12.82; *SD* = 1.22);
- 10 HFA teenagers (9 boys) of 10 years and 2 months to 17 years in age (*M* = 13.31; *SD* = 2.04);
- 10 typically developing (TD) control teenagers (2 boys) of 10 years and 8 months to 14 years and 1 month in age (*M* = 12.75; *SD* = 1.20).

The teenagers with DLD presented a specific clinical profile, i.e., disturbed oral language in production without comprehension disorder and preserved cognitive skills. All participants with DLD had been diagnosed by a multidisciplinary team (a child psychiatrist, neuropsychologist, and speech therapist) at a referral center for language and learning disorders. The two criteria for inclusion in the DLD group were as follows:

- First, a score of less than least −1.25 standard deviations from the mean on the two standardized tests used during the speech and language therapy assessment:
  ∘ The Computerized Oral Language Assessment (BILO-3C; Khomsi et al., 2007) calibrated for 7–16 years of age;
  ∘ The battery of Oral and Written Language, Memory, Attention (L2MA; Chevrie-Muller et al., 1997) calibrated for 8–11 years of age;
- Second, an Intelligence Performance Quotient (IPQ) of above 80 on the psychometric intelligence assessment battery (WISC III; Weschler, 1996) used during the neuropsychological assessment.

The diagnosis of DLD met the criteria for Expressive Language Acquisition Disorder (F80.1) as defined in the World Health Organization’s ICD-10 classification of mental and behavioral disorders of children and teenagers (World Health Organization, 2001). As we were interested by the narrative skills of teenagers with DLD in language production situation, we only selected participants with an expressive language acquisition disorder (Rapin and Allen, 1983). In addition, the participants with DLD had no neurological, sensory, relational, or educational disorders and were all enrolled in middle school.

Teenagers with HFA presented a specific clinical profile as defined by Vermeulen (2013), namely, stereotyped behaviors, restricted interests, problems with social interactions and hyper or hyposensitivity to the environment. All participants with HFA had been diagnosed by a multidisciplinary team (a child psychiatrist, neuropsychologist, and speech therapist) in a Belgium referral center for autism. The diagnosis of HFA met the criteria for neurodevelopmental disorders affecting communication skills as defined in the DSM-V (American Psychiatric Association, DSM-5 Task Force, 2013). In addition, teenagers with HFA included in this study did not show delays in cognitive development. As such, these participants were considered to have HFA (Rogé, 2015). This inclusion criterion was measured by individual performance on the Matrices test, which is part of the Perceptual Reasoning index of the WISC-IV (Weschler, 2005) and is considered a good measure of fluid intelligence (Grégoire, 2010). The average T-score obtained from the non-verbal WISC matrices was 45.90 for the HFA group. Six of the HFA participants were enrolled in a full-time school for HFA students in Belgium and were recruited by their teachers who had seen an advertisement broadcast during the screening of a television program. The other four HFA participants contacted the experimenters directly after viewing an advertisement posted on social networks.

Typical French-speaking Belgian teenagers were recruited by the authors through several social networks. These teenagers were all enrolled in mainstream school and did not have any language or cognitive disorders.

### Group Matching

The three groups were matched on chronological age. The within-group variances for the chronological ages of the participants were homogeneous (Levene test: *p* = 0.13). Participants with HFA and TD participants were matched on non-verbal IQ. The average T-score obtained from the non-verbal WISC matrices was 53.57 for the TD group and 45.90 for the HFA group. The *U* of Mann-Whitney showed that there was no significant difference (*U* = 35.50; *p* = 0.074). Participants with DLD and HFA were also matched by gender. In addition, the participants in each group of DLD and HFA were enrolled in schools in the same district, which ensured a similar parental socioeconomic status.

### Narrative Task

The narrative task was inspired by the Spencer Project “Developing Literacy in Different Contexts and Different Languages, 1997–2001” (Berman and Verhoeven, 2002; Berman et al., 2002; Berman, 2005). It assesses the ability to produce a narrative in a communicative context of telling of a personal experience to someone who does not know the event and needs to be told about it. The experimenter asked the participants to tell a story about a school robbery or quarrel that they had either experienced or witnessed one day. The narrative of personal event produced by the teenagers were often related to everyday school life, such as thefts of pens or mobile phones and quarrel between friends or about a game in the playground. When teenagers did not have a story to tell about a robbery or a quarrel that had happened at school, they could tell one that had happened elsewhere. The experimenter asked the participants to be as specific as possible so that she could understand what had happened. Once the participants agreed to do so, they began to tell their stories, which the experimenter recorded. Examples 1, 2, and 3 illustrate what the participants produced.

Example 1: J., 12 years 10 months, boy, teenager with DLD

*“Un sour ma sæur elle avait emmené son MP3. Et au collège ce on a. Elle avait laissé dans son sac. Pendant la récré en a un qui l’a pris. Et l’aute y disait que c’était pas lui. Et ma sæur elle disait que c’était lui parce qu’elle l’avait vu. Et après à lui il avait il le MP3 de ma sæur. Il l’a après il l’a donné au prof et il avait dit que c’était à lui. Mais c’était à ma sæur. Et il l’a récupéré. Ma sæur elle l’a même pu”*
Translation in English in understandable form: *“One day, my sister she brought her MP3 to middle-school. And at the middle-shool we have. She left in her bag. During the playtime there is one who took it. And another said it wasn’t him. And my sister she said it was him because she saw him. And then, him he had my sister’s MP3. He, after, he gave it to the teacher and he said it was his. But it was my sister’s. And it got it back. My sister she doesn’t even have it anymore.”*

Example 2: T., 14 years 11 months, boy, teenager with HFA

*“C’est l’histoire de Marc et Michael. Ils sont tous les deux dans un snack. Ils ont commandé tous les deux des frites avec. Y en a un qui. Ils ont pris une sauce pour deux, pour tous les deux. Puis Marc, je sais plus comment il s’appelle, il prend toute la sauce alors que Michael en voulait. Alors ils se sont disputés pour ça. Et puis après ça a recommencé pour une deuxième fois. NEN NEN EN. Et puis encore après, 5 jours dans le même snack.”*
Translation in English in understandable form: *“This is the story of Marc and Michael. They are both in a snack. They both ordered fries with. There is one who. They took a sauce for two, for both of them. Then Marc, I don’t know anymore what his name is, he takes all the sauce when Michael wanted some. So they quarrelled about it. And then it started again for a second time. NEN NEN EN (arbitrary vocalizes). And then again after 5 days in the same snack.”*

Example 3: C., 12 years 11 months, boy, Typically Developing teenager.

*“Quand on allait manger au self en général on laissait nos sacs tous en tas contre un mur dans la cour et moi j’avais laissé mon portable dedans comme beaucoup de personnes laissaient des affaires de valeur des fois on pensait qu’on pouvait avoir confiance qu’on n’était pas beaucoup dans le collège. Et donc j’laisse mon portable donc on allait manger et en r’venant j’me suis rendue compte quelques minutes après qu’j’avais pu mon portable. Donc on a d’mandé un peu autour si y’en avait qu’avait vu et on a appris un peu, y’en a qui voulait pas trop balancer ou dire c’que leurs copains avaient fait mais on a appris que y’en avait pas mal des élèves qui fouillaient dans les sacs pendant la pause du midi. Mais on a jamais su qui c’était et j’ai jamais retrouvé l’portable. Alors d’abord j’l’ai signalé au collège mais le problème c’est que dans le règlement intérieur normalement on n’a pas le droit d’en avoir sur soi et qu’y sont pas responsables des vols donc. Mais sinon j’ai prévenu mon opérateur pour bloquer le portable et ma mère a fait une déclaration de vol au commissariat.”*
Translation in English in understandable form: *“When we went to eat in the cafeteria, we usually left our bags in a pile against a wall in the playground and I left my cell phone in it, as many people leave valuable things behind, sometimes we thought we could trust that there weren’t many of us in the school. And so I leave my cell phone so we go to eat and when I came back I realized a few minutes later that I didn’t have my cell phone anymore. So we asked around if there were any who had seen and we learned a little, there were some who didn’t want to denounce too much or say what their friends had done, but we learned that there were quite a few students who were rummaging in the bags during lunch break. But we never knew who it was and I never found the cell phone. So first I reported it to the school but the problem is that in the rules of procedure you are not allowed to carry them and they are not responsible for theft so. But otherwise I notified my operator to block the phone and my mother made a theft report to the police station.”*

### Procedure

All narratives were collected with the parents’ consent. All stories were recorded on a voice recorder placed in front of the adolescent. The interviews during which the stories were collected lasted on average 30 min.

### Coding

Narratives were transcribed with “CHILDES” (Child Language Data Exchange System) software, which allows the study of spontaneous language in natural situations and the qualitative and quantitative analysis of discourse (MacWhinney, 2000). The CHAT (Codes for the Human Analysis of Transcripts) command was used to code narratives produced by the teenagers on different linguistic indices: coherence, cohesion, and social-cognitive skills (MacWhinney, 2000). In addition, length of the narratives (in number of words) was considered beforehand to ensure that the narratives produced by the teenagers with DLD and with HFA were comparable. Two final year speech therapy students coded all the narratives produced by the participants. They were supervised by a lecturer in psychology who validated their coding.

#### Story Length

Story length was measured by three variables:

– The number of words produced. For instance, in J.’s narrative (see Example 1) we counted 93 words.
– The number of utterances produced. For this count, the definition of an utterance including a conjugated verb, or an infinitive verb was applied. For instance, in T.’s narrative (see Example 2) we counted 11 utterances:
  *“This* ***is*** *the story of Marc and Michael. / They* ***are*** *both in a snack bar. / They* ***are*** *both* ***ordered*** *fries with./ They* ***took*** *a sauce for two, for both of them. Then Marc, / I* ***don’t know*** */ what his name* ***is****/, he* ***takes*** *all the sauce / when Michael* ***wanted*** *some./ So they* ***quarreled*** *about it./ And then it* ***started*** *again for a second time./ NEN NEN EN. And then again after 5 days in the same snack bar”*
– The average length of utterances corresponded to the mean number of words per utterances. For instance, in C.’s narrative (see Example 3) we counted 194 words, 39 utterances and then an average length of 5 words per utterance.

#### Narratives Skills

##### Coherence

First, ***coherence*** of the narratives was evaluated using a grid adapted from Schelstraete (2011) that assessed respect to the narrative schema:

- The initial situation. The participant has to set out the initial situation (“once,” “one day”) and/or to introduce the characters, a location, etc. The initial situation was coded on 2 points: 0 point was given when no element related to the initial situation was present; 1 point was given when there was just one element; and 2 points were given when there were at least two elements.
- The disruptive element. At this step, a theft or a quarrel should be presented in the narrative. The disruptive element was coded on 1 point: 0 point when it did not happen and 1 point when it happened.
- The complication. This step is composed of 3 sub-steps:
  - *The reaction or the internal response*: what the main character says or thinks in response to the disruptive element;
  - *The goal*: what the main character decides to do;
  - *The attempt*: the effort of the main character to solve the problem.
  One point was given for each sub-stage present in the narrative (max. score = 3).
- The resolution (whether it is successful or not). The resolution was coded on 2 points: 0 point was given when there was no resolution; 1 point when there was a resolution but the participant did not explain why it was successful or unsuccessful, and 2 points were given when there was a resolution and when the participant explained why it was successful or not.

The maximum number of points that could be obtained was 8. For instance, in C.’s narrative (see Example 3) we counted a total of 7 points obtained as such:

Initial situation: *“When we went to eat in the cafeteria, we usually left our bags in a pile against a wall in the playground and I left my cell phone in it, as many people leave valuable things behind, sometimes we thought we could trust that there weren’t many of us in the school”:* 2 points.
The disruptive element: “*when I came back I realized a few minutes later that I didn’t have my cell phone anymore”:* 1 point.
The complication: “*So we asked around if there were any who had seen and we learned a little, there were some who didn’t want to denounce too much or say what their friends had done, but we learned that there were quite a few students who were rummaging in the bags during lunch break. But we never knew who it was and I never found the cell phone”:* 3 point.
The resolution*: “So first I reported it to the school but the problem is that in the rules of procedure you are not allowed to carry them and they are not responsible for theft so. But otherwise I notified my operator to block the phone and my mother made a theft report to the police station”:* 1 point.

##### Cohesion

Second, ***cohesion*** of the narratives was assessed using a grid adapted from Favart et al. (2016) by considering the presence of anaphora and connectors.

##### Anaphora

First, the total number of correct anaphora produced per word were counted (see Table 1 for examples). Second, the number of different anaphora produced were counted and divided by the number of total anaphors.

**TABLE 1:** Linguistic elements (bold provided) coded as correct anaphors.

Linguistic elements	Examples
Determiner + noun	I saw Max. **This boy**
Determiner + noun (+ adjective)	The older sister
Determiner + noun + relative	The boy **who**
Determiner + noun + pronoun	The teacher **she**
Pronoun	The girl sees **him**
Proper noun	Hugo

For instance, in J.’s narrative (see Example 1) we counted a total of 17 anaphora and a total of 5 different anaphora:

*“One day, my sister* ***she*** *brought her MP3 to middle-school. And at the middle-shool we have.* ***She*** *left* ***it*** *in her bag. During the playtime there is one* ***who*** *took* ***it***. *And another one said it wasn’t* ***him***. *And my sister* ***she*** *said it was* ***him*** *because* ***she*** *saw* ***him***. *And then*, ***him*** ***he*** *had my sister’s MP3.* ***He***, *after, he gave* ***it*** *to the teacher and* ***he*** *said it was his. But it was my sister’s. And it got* ***it*** *back. My sister doesn’t even have* ***it*** *anymore.”*

Note that when a participant used “they” to name several protagonists, the anaphora was counted for each protagonist. For example, in “they (Hugo and Mattéo) were in the yard,” the anaphora was counted as 1 for Hugo and 1 for Mattéo. Furthermore, in the case of a repetition of a part of the statement, i.e., a repetition that corresponds to an element of speech, the anaphora was counted only once. For example, in “the boy, the boy, sees,” “the boy” was counted only once as an anaphora.

##### Connectors

The total number of correct connectors used correctly per utterance was recorded as well as the number of different connectors, divided by the total number of connectors. The following several types of connectors were recorded:

- Spatial. For example: “down,” “up,” “where.”
- Temporal. For example: “when,” “now,” “suddenly,” “after,” “then,” “during,” “while.”
- Purpose. For example: “so that.”
- Causal. For example: “because.”
- Consequence. For example: “thus,” “therefore,” and “in order to.”
- Conditional. For example: “if,” “provided that,” and “in case.”
- Coordinating conjunctions. For example: “but,” “and,” “therefore,” and “because.”

In T.’s narrative (see Example 2), we counted a total of 4 connectors and a total of 3 different connectors.

*“This is the story of Marc and Michael. / They are both in a snack bar. / They are both ordered fries with./ They took a sauce for two, for both of them.* ***Then*** *Marc, / I don’t know / what his name is/, he takes all the sauce /* ***when*** *Michael wanted some./* ***So*** *they quarreled about it./* ***And then*** *it started again for a second time./ NEN NEN EN.* ***And then*** *again after 5 days in the same snack bar”*

Note that the connector “*and then*” was counted as one connector and as a different connector than “**then**.”

#### Social-Cognitive Skills Used in Narratives

Based on Reilly et al. (1990), two types of social-cognitive skills were identified from the narratives produced by teenagers. On the one hand, we counted positive social-cognitive elements relating to “emotions and cognition,” i.e., referring to the characters’ affective and cognitive mental states. These include statements such as “*my classmate was sad*” and “*at that moment I think that*.” On the other hand, we noted negative social cognitive elements such as voice noises (arbitrary vocalizations) which decrease the quality of the interaction. For each category, the total number of social-cognitive elements produced per word was recorded. For instance, in T.’s narrative (see Example 2) we counted 3 voice noises:

*“This is the story of Marc and Michael. They are both in a snack bar. They are both ordered fries with. They took a sauce for two, for both of them. Then Marc, I don’t know what his name is, he takes all the sauce when Michael wanted some. So they quarreled about it. And then it started again for a second time.* ***NEN NEN EN***. *And then again after 5 days in the same snack bar”*

## Results

### Length of Narratives

To control for length of the narratives, we compared the total number of words produced per narrative, the total number of utterances produced per narratives, and the average length of utterances produced in the narratives. The different dependent variables were analyzed with ANOVAs using JASP software. Results at the 0.05 level were considered significant. These three variables were analyzed using a one-factor analysis of variance with group (DLD vs. HFA vs. TD) as a between-participants factor.

The total number of words produced per narrative, the total number of utterances produced per narratives, and the average length of utterances produced in the narratives (see Table 2) did not significantly differ between the three groups, respectively, *F*(2, 27) = 2.49, *p* = 0.10; *F*(2, 27) = 2.01, *p* = 0.15; *F*(2, 27) = 2.88, *p* = 0.07.

**TABLE 2:** Means (M) and standard deviations (*SD*) of number of words, number of utterances, and average length of utterances produced in narratives by each group.

	Groups		
Variables	DLD	HFA	TD
Total number of words	68 (*27*)	164 (*159*)	332 (*433*)
Total number of utterances	8 (*3*)	14 (*7*)	17 (*15*)
Average length of utterances	9 (*4*)	11 (*6*)	15 (*7*)

### Narrative Skills

#### Coherence

Given the characteristics of the coherence data, non-parametric statistical tests were conducted. The different dependent variables were analyzed with Krsukal-Wallis test using JASP software. Results at the 0.05 level were considered significant.

**FIGURE 1:**
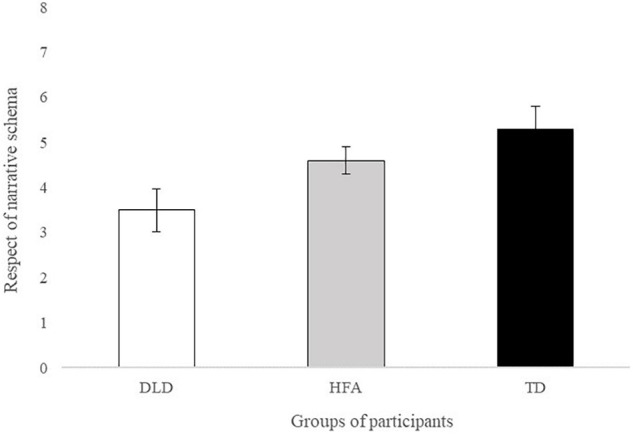
Respect of narrative schema in the narratives of DLD, HFA and TD participants. Error bars represent standard errors.

First, the analysis compared the total number of points obtained in the respect of narrative framework (max = 8 points). A Kruskal-Wallis test indicated a significant group difference [*χ^2^*(2) = 6.00, *p* = 0.05, *ε^2^* = 0.21]. *Post-hoc* Wilcoxon tests revealed significant difference between participants with DLD (*M* = 3.5; *SD* = 1.5) and TD participants (*M* = 5.3; *SD* = 1.6), *W*(2) = 3.22, *p* = 0.05. The differences between participants with DLD and participants with HFA (*M* = 4.6; *SD* = 0.97) and between participants with HFA and TD participants were not significant [respectively: *W*(2) = 2.59, *p* = 0.16; *W*(2) = 0.85, *p* = 0.82] (see Figure 1).

We further analyzed which steps were successful (or not) in the different groups. The analyses compared the total number of points obtained in each step (initial situation: max. score = 2; disruptive element: max. score = 1; complication: max. score = 3; resolution: max. score = 2). The variables were again analyzed with a Kruskal-Wallis test to compare the three group (DLD vs. HFA vs. TD) in each step (see Table 3). For the initial situation, disruptive element and resolution steps, the analyses did not indicate significance difference between the groups [initial situation, *χ^2^*(2) = 4.11, *p* = 0.13, *ε^2^* = 0.14; disruptive element, *χ^2^*(2) = 0.56, *p* = 0.76, *ε^2^* = 0.02; and resolution, *χ^2^*(2) = 1.59, *p* = 0.45, *ε^2^* = 0.05]. However, for the complication step, an effect of the group was observed, *χ^2^*(2) = 7.00, *p* = 0.03, *ε^2^* = 0.24. *Post hoc* Wilcoxon tests indicated a significant difference between participants with DLD and TD participants, *W*(2) = 3.36, *p* = 0.04. In addition, the difference between participants with DLD and participants with HFA or between participants with HFA and TD participants were not significant [respectively, *W* (2) = 2.59, *p* = 0.16, and *W* (2) = 1.60, *p* = 0.49].

**TABLE 3:** Mean (M) scores and standard deviations (*SD*) for each narrative step in the three groups.

	Groups		
Variables	DLD	HFA	TD
Initial situation	1.10 (*0.57*)	1.50 (*0.53*)	1.60 (*0.52*)
Disruptive element	0.80 (*0.42*)	0.90 (*0.32*)	0.90 (*0.32*)
Complication	0.80 (*0.63*)	1.30 (*0.48*)	1.70 (*0.82*)
Resolution	0.70 (*0.67*)	0.80 (*0.42*)	1.10 (*0.88*)

#### Cohesion

The different dependent variables were analyzed with ANOVAs using JASP software. Results at the 0.05 level were considered significant. A one-factor analysis of variance, with group of participants (DLD vs. HFA vs. TD) as a between-participant factor was carried out on the variables related to cohesion. Mean and standard deviations of anaphora and connectors produced in each group of participants are indicated in Table 4.

**TABLE 4:** Means (M) and standard deviations (*SD*) of anaphors and connectors produced by each group of participants.

	Groups		
Variables	DLD	HFA	TD
Number of anaphors per word	0.14 (*0.07*)	0.13 (*0.04*)	0.14 (*0.07*)
Number of different anaphors	0.66 (*0.25*)	0.5 (*0.22*)	0.52 (*0.52*)
Number of connectors per utterance	0.82 (*0.49*)	0.83 (*0.69*)	1.06 (*0.76)*
Number of different connectors	0.69 (*0.23*)	0.65 (*0.26*)	0.61 (*0.16*)

##### Anaphora

A first analysis compared the number of correct anaphors produced per words in the narratives. There was no significant difference between groups, *F*(2, 27) = 0.15, *p* = 0.86. A second analysis compared the number of different anaphors. This analysis did not show difference between groups, *F*(2, 27) = 1.67, *p* = 0.21.

##### Connectors

A first analysis compared the number of correct connectors produced per utterance in the narratives. The group effect was not significant, *F*(2, 27) = 0.42, *p* = 0.66. A second analysis compared the number of different connectors. The group effect was not significant, *F*(2, 27) = 0.38, *p* = 0.69.

#### Social-Cognitive Skills

##### Emotions-Cognition

The different dependent variables were analyzed with ANOVAs using JASP software. Results at the 0.05 level were considered significant. A one-factor analysis of variance with group (DLD vs. HFA vs. TD) as a between-participant factor was carried out on percentage of emotion-cognition items produced per word in the narratives. The group effect was not significant, *F*(2, 27) = 0.43, *p* = 0.65 (see Table 5).

**TABLE 5:** Means (M) and standard deviations (*SD*) of number of emotions/cognition words produced per word and number of voice noises produced per word in each group.

	Groups		
Variables	DLD	HFA	TD
Number of emotion/cognition per word	0.003 (*0.008*)	0.002 (*0.005*)	1.006 (*0.76)*
Number of voice noises per word	0	0.007 (*0.011*)	0

##### Arbitrary Vocalizations (Voice Noises)

No statistical analysis was conducted since only three HFA teens produced arbitrary vocalizations (see Table 5).

## Discussion

The present study investigated the narrative skills of teenagers with DLD and HFA while engaged in a communicative ecological situation involving the telling of a personal experience. The aim of the study was to highlight the narrative and social-cognitive skills of these teenagers based on their ability to be coherent and cohesive and to produce positives or negative social-cognitive elements. The aim was also to highlight which difficulties exist persist in adolescence to better interpret the language similarities and differences between the two types of disorders. As we hypothesized, the results obtained show difficulties in coherence only for teenagers with DLD in the complication step. Moreover, the second hypothesis is not verified since the results obtained show that, in narrative of personal event, teenagers with HFA no longer have difficulties with social-cognitive skills. Only three of them still produced arbitrary vocalizes (voice noises) which hindered communication while producing the narratives.

More specifically, DLD teenagers followed the narrative schema less well than teenagers with HFA and typical teenagers. This first set of results is consistent with the literature (for DLD: Reed et al., 2007; Petersen, 2011; for HFA: Crane et al., 2010; Barnes and Baron-Cohen, 2012). In adolescence, participants with DLD have difficulties with coherence in the complication step. Uzé and Stonehouse (1996) attribute this difficulty with adhering to the narrative schema to the fact that children with DLD remain focused on the present and do not access the spatiotemporal distance needed to provide accounts of personal experience. Furthermore, the complication step which contains three sub-steps requires narrators to develop their arguments. DLD teenagers who have specific language difficulties in production encountered difficulties to produce arguments in each sub-tests of the complication. It seems that this difficulty exists in adolescence among participants with DLD.

Regarding cohesion, i.e., the number of anaphors and connectors produced in accounts of personal experiences and the variety of anaphors and connectors used, the findings do not show differences between the teenagers with DLD, those with HFA and the typical teenagers. This second set of results does not support the hypothesis that there are difficulties in cohesion for teenagers with DLD. These results could be explained by the task chosen in this study. Indeed, when telling a personal experience, participants with DLD, particularly teenagers, mobilize the extent of their linguistic skills to maximize their chance of being understood by their interlocutor orally (Broc et al., 2016) but also in writing (Broc et al., 2013).

Finally, this study shows that in adolescence, participants with HFA do not have difficulties in social-cognitive skills, as those with DLD or with typical development. There was no difference between the three groups in positive social-cognitive skills (production of emotions and cognition). Only three teenagers with HFA produced arbitrary vocalizes (noise voices).

## Conclusion

The present study showed that narrative and social-cognitive skills do not appear to differ between teens with DLD or HFA and TD teens. In both these groups, cohesion of their narratives was comparable to that of typical participants of the same ages. However, teenagers with DLD still had difficulties with producing a discourse that was fully coherent particularly in the complication step of the narrative schema. Finally, the (positive and negative) social-cognitive skills of teenagers for both DLD and with HFA were comparable to those of typically developing teens. The narrative of personal event allows them to mobilize the extent of their linguistic skills and maximize their chance of being understood by their interlocutor.

Two limitations of this study should be noted. The lack of comparison between children and teenagers with DLD and HFA did not allow for the analysis of the development of narrative and social-cognitive skills. Yet, such a comparison is necessary to define benchmarks for the development of narratives and social-cognitive skills between DLD and HFA. Future studies should therefore take a broader developmental perspective, from childhood to adulthood, to examine how the narrative and social-cognitive difficulties observed in DLD and HFA change at different ages. This will also help practitioners to better target the remediations and interventions.

The second limitation is related to the assessment of social-cognitive skills, which was limited in this study. We indeed only considered items related to the presence of linguistic devices indicating emotions and cognition and voice noises (arbitrary vocalizations). Considering more elements related to the social-cognitive skills should help to highlight specific social-cognitive difficulties, particularly in teenagers with HFA. For example, the assessment of the theory of mind, the ability to attribute mental states to oneself and to others (Baron-Cohen, 1991), could identify specific difficulties. The Winner and Perner’s test of children’s understanding of *false belief* is an appropriate way to assess the theory of mind (Wimmer and Perner, 1983; Baron-Cohen, 2000).

The findings of the study have practical and clinical implications. Practitioners managing speech therapies for children with language impairments should pay more attention to skills related to high-level language activities such as coherence and cohesion performance in narrative production. Language difficulties are usually assessed by analyzing formal aspects of language production (phonology, morphology, syntax) in standardized tasks that require for example word or sentence repetition. Previous studies conducted both in oral and written language (Broc et al., 2013; Broc et al., 2016) have shown that using ecological language production provided others information language difficulties. This study has shown indeed that an ecological language production situation that requires telling a personal narrative bring to the light specific language difficulties related to the respect of the narrative schema. Therefore, future studies should include such ecological language situation and use more diverse measures of narrative skills in adolescents with DLD.

## Data Availability Statement

The raw data supporting the conclusions of this article will be made available by the authors, without undue reservation.

## Ethics Statement

Ethical review and approval was not required for the study on human participants in accordance with the local legislation and institutional requirements. Written informed consent to participate in this study was provided by the participants’ legal guardian/next of kin.

## Author Contributions

All authors contributed equally to the writing of the manuscript.

## Conflict of Interest

The authors declare that the research was conducted in the absence of any commercial or financial relationships that could be construed as a potential conflict of interest.

## Publisher’s Note

All claims expressed in this article are solely those of the authors and do not necessarily represent those of their affiliated organizations, or those of the publisher, the editors and the reviewers. Any product that may be evaluated in this article, or claim that may be made by its manufacturer, is not guaranteed or endorsed by the publisher.
